# Thrombin kinetics determines robust plasma clot architecture

**DOI:** 10.1016/j.rpth.2026.106884

**Published:** 2026-08-03

**Authors:** François Caton, Landry Seyve, Carhel Dassi, Denis Roux, Benoît Polack

**Affiliations:** 1 Université Grenoble Alpes, Laboratoire Rhéologie et Procédés, UMR CNRS 5520, Grenoble, France; 2 Université Grenoble Alpes, Laboratoire TIMC, UMR CNRS 5525, Grenoble, France

**Keywords:** fibrin clot structure, fibrinography, robustness, thrombin generation, confocal microscopy

## Abstract

**Background:**

Altered fibrin clot architecture is a distinct signature in cardiovascular diseases. To determine the origin and importance of these alterations, a fundamental understanding of normal plasma clotting mechanisms is essential. While biochemical and structural aspects of fibrin formation are individually well documented, their mechanistic link lacks a quantitative description.

**Objectives:**

This study addresses two questions: How does fibrin architecture vary with tissue factor concentration ([TF]) in normal plasma, and can a simple mechanistic relationship between fibrin structure and thrombin generation be extracted?

**Methods:**

The structuring of clots formed in TF-triggered normal pooled plasma and IIa-triggered fibrinogen was compared by varying the trigger concentrations. Architecture was quantified using confocal microscopy (pore size) and fibrinography (nanostructure). Thrombin kinetics were monitored using thrombinography.

**Results:**

Although purified and plasma clots appeared morphologically identical, their ultrastructural parameters showed fundamentally different sensitivities to trigger concentrations. In purified systems, a 100-fold [IIa] increase induced a 30-fold decrease in protofibril number (*N*_Final_) and a 5-fold change in pore size. Conversely, plasma clots exhibited remarkable robustness: a 40-fold increase in [TF] resulted in only a 2-fold change in *N*_Final_, with little pore size evolution. Crucially, by applying inverted purified data to plasma structural data, we predicted thrombin concentrations during the exponential growth phase of plasma calibrated automated thrombinography curves.

**Conclusions:**

Our findings show that the unique functional shape of the thrombin burst, rather than the trigger concentration, governs plasma fibrin architecture. This inherent robustness supports the existence of a “normal” clot structure and the potential of fibrinography as a robust structural biomarker candidate.

## Essentials

• The link between the chemical reactions that form fibrin and the structure of plasma blood clots is little known.
• Clot formation in plasma was compared with clot formation from purified fibrinogen while varying the concentrations of clotting triggers.
• Unlike clots formed from purified fibrinogen and thrombin, clots formed in plasma changed very little when the concentrations of clotting triggers were varied.
• This robust clot formation appears to result from the characteristic rise of thrombin production over time during clotting, rather than simply from the total amount of thrombin produced.

## Graphical abstract

**Figure:**
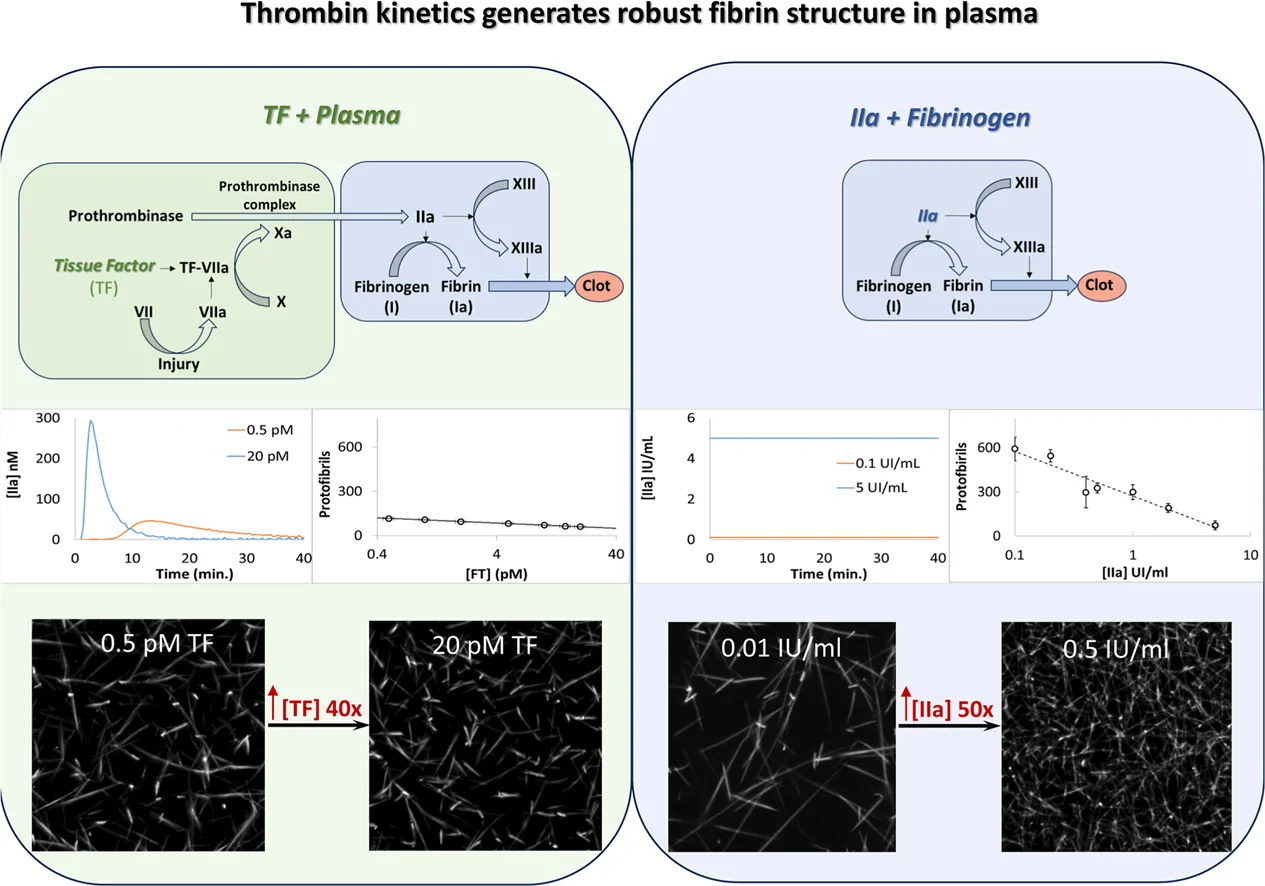

## 1 Introduction

The structure of the fibrin clot at different scales (architecture hereafter) is a key element in blood coagulation, as it is responsible for the mechanical stability of the blood clot with respect to flow, platelet-dependent clot retraction, and overall mechanical stresses [[1], [2], [3]]. Fibrin clots also play an important role in tissue reconstruction, as new tissue is built on the fibrin scaffold [4]. Finally, the fibrin clot structure must be amenable to proper disintegration after the injury has been repaired. In the last decade, a significant number of studies have reported altered structure and lysis of fibrin clots in patients with a wide range of cardiovascular diseases [[5], [6], [7], [8]]. These fibrin clots were more compact and resistant to lysis than those in controls, as determined by permeation, turbidity, and lysis assays.

To appraise the origin and importance of these changes, a fundamental understanding of the mechanisms of clotting in normal plasma is necessary [9]. Qualitatively, the construction of the fibrin clot structure starts with the release of tissue factor (TF) at the site of injury, which, through a complex cascade of biochemical reactions, leads to the formation, burst, and regulation of thrombin (noted IIa where relevant). Thrombin cleaves fibrinogen fibrinopeptides to allow for the formation of the fibrin network. In the last century or so, there has been considerable work on the modeling and experimental measurement of the biochemistry of plasma coagulation. Indeed, most investigations on the effect of TF concentration have dealt with the measurement or modeling of the clotting biochemical cascade [10,11]. Current modeling of the clotting cascade is described by up to 81 coupled differential equations [[12], [13], [14]] and is relatively competent in predicting thrombin generation curves [15] measured in plasma by methods such as calibrated automated thrombinography (CAT) [[16], [17], [18]]. Concerning the structural aspects of fibrin formation, major progress has been made in understanding the different steps of purified fibrinogen clotting with purified thrombin [[19], [20], [21], [22], [23], [24], [25], [26]] as well as the early stages of TF-clotted plasma [27].

However, to our knowledge, the understanding of the *interaction* between the biochemical cascade and the formation of fibrin structure is mostly qualitative. For instance, the influence of TF concentration—the physiological trigger—on the structural characteristics of plasma clots is poorly understood. To our knowledge, there is only one quantitative study that investigated the influence of TF concentration on the turbidity of plasma clots. The study showed a progressive decrease in the maximum turbidity with increasing TF concentration [11]. Conversely, the effect of various concentrations of added exogenous thrombin in plasma showed larger changes in fibrin structure [28,29]. However, as He et al. [30] recently stated in plasma assays using purified thrombin, fibrin network porosity is regulated by both the added thrombin and the subsequent positive feedback generated by thrombin through factor XI. So, thrombin cannot be used as a reference trigger in plasma for the specific purpose of characterizing the effect of thrombin generation kinetics on fibrin architecture. In the range where feedback contamination is nonnegligible (0–0.5 IU/mL), the actual IIa experienced by fibrinogen is undefined and cannot be measured with current thrombinography methods. Conversely, the dependence of plasma clot structure on exogenous thrombin concentration has been well studied and remains relevant for characterizing, for example, fibrinogen-dependent structural properties [28,29,31].

This study addresses two main questions. First, how does fibrin architecture vary with [TF] in normal pooled plasma? Second, is it possible to extract a simple mechanistic relationship between fibrin structure and thrombin generation in plasma? To help understand the interaction between the biochemical cascade and the structuring of fibrin formation, a comparative model system is required. Given the above arguments, we chose to use the simplest clotting model as a reference, one with monodisperse fibrinogen clotted with varying quantities of purified thrombin. The architecture of the plasma and purified clots was determined using confocal microscopy for the microstructure (pore size), fibrinography for the nanostructure, and thrombin evolution was determined using CAT.

## 2 Methods

### 2.1 Materials and reagents

A frozen normal plasma pool was obtained from Cryopep with a Clauss fibrinogen concentration of [Fg] = 2.7 g/L. All experiments were performed using this plasma pool. Throughout this study, triplicates represented technical replicates with three independent measurements from the same pooled plasma preparation.

The CAT reagents (MP-Reagent and PPP-Reagent High), thrombin calibrator, and FluCa-Kit (FluoBuffer and FluoSubstrate) were obtained from Diagnostica Stago. The different TF-containing solutions used for all plasma experiments were dilutions of the MP reagent (0 pM TF) and PPP Reagent High (20 pM TF), both containing the same concentration of phospholipids. Multiplates (Immulon 2HB) were obtained from Thermo Fisher Scientific. Monodisperse purified fibrinogen (Clottafact; Mw/Mn < 1.01, aggregate-free, as determined by size-exclusion chromatography–multiangle light scattering [26]) was obtained courtesy of Laboratoire Français du Fractionnement et des Biotechnologies, Les Ulis, France. The fibrinogen concentration in all purified samples was 3 g/L.

### 2.2 Thrombin generation test

Thrombin generation was measured as previously described [32] using a CAT fluorimeter (Diagnostica Stago). Clotting was initiated by automatically dispensing the fluorogenic substrate (Z-Gly-Gly-Arg-AMC) in CaCl_2_ (416 and 16.7 mM, final concentrations, respectively), calibrated against wells containing α2-macroglobulin–thrombin complex and plasma, and analyzed using Thrombinoscope software (Thrombinoscope BV), according to Hemker’s method [16,17]. The final TF and phospholipid concentrations were 2 pM and 4 mM, respectively, and all assays were performed in triplicate from the same plasma pool. Thrombin generation was monitored using excitation and emission wavelengths of 390 and 460 nm, respectively, and recorded for 60 minutes at 37 °C.

### 2.3 Confocal microscopy and pore size measurements

Fibrinogen was labeled at 1/10 V/V with ATTO 488 (ATTO-TEC GmbH), according to the manufacturer’s instructions. Coagulation was performed as described in the thrombin generation assays (see above). Three-dimensional (3D) stacks of the clots were imaged using a Zeiss LSM710 confocal microscope with a 63×/1.2 water-immersion objective (Zeiss). To visualize the stacks, we used Smooth Manifold Extraction (SME), which is an objective 2D projection [33]. The pore size was determined using the bubble method on the complete stacks (see ref. [26] for details).

### 2.4 Fibrinography

Fibrinography was used as the structural assay, as previously described [32]. It uses the same reaction conditions and volumes as CAT, only omitting the fluorophore. The fluorophore volume (20 μL) was replaced with an equivalent volume of buffer to maintain identical final concentrations. The aim of the assay was to determine the evolution of the nanostructure of the fibrin clot instead of thrombin generation.

To that end, spectra between 500 and 820 nm (2.5 nm resolution) were acquired every 6 seconds during 30 minutes at 37 °C (±0.5 °C) in a SPECTROstar Omega microplate reader (BMG Labtech). From each spectrum, the average number of protofibrils per fiber was determined using the “corrected Yeromonahos method” [26,32], which has been shown to be in good to excellent agreement with atomic force microscopy measurements [28], stochastic optical reconstruction microscopy [34], and electron microscopy [29]. Significant deviations are expected for very thin (<100 nm) or very thick fibers (>300 nm) [34]. However, because the same method was used to compare the purified and plasma systems, these deviations were systematic errors and were therefore of little importance. All assays were performed in triplicate using the same plasma pool.

### 2.5 Fibrinography and thrombin generation test characteristic parameters

The fibrinography curves are sigmoidal (see Figure 1), which are usually well represented by logistic equations. Therefore, three parameters should be sufficient to characterize them: the time at maximum velocity (*T*_*V*max_), the maximum velocity (*V*_max_), and the maximum number of protofibrils (*N*_max_). However, particularly in plasma, there is often a slow, log-like temporal evolution of the number of protofibrils after most of the structuring has occurred (Figure 3, left panel). Consequently, we defined the time of completion of the reaction as *T*_Final,_ where the reaction velocity was less than 3% of *V*_max_, and the associated effective maximum number of protofibrils was denoted as *N*_Final_. For comparison with the literature, the usual clotting time *T*_c_ was also calculated, which is the extrapolation to the abscissa of the tangent of the maximum velocity.

**Figure 1:**
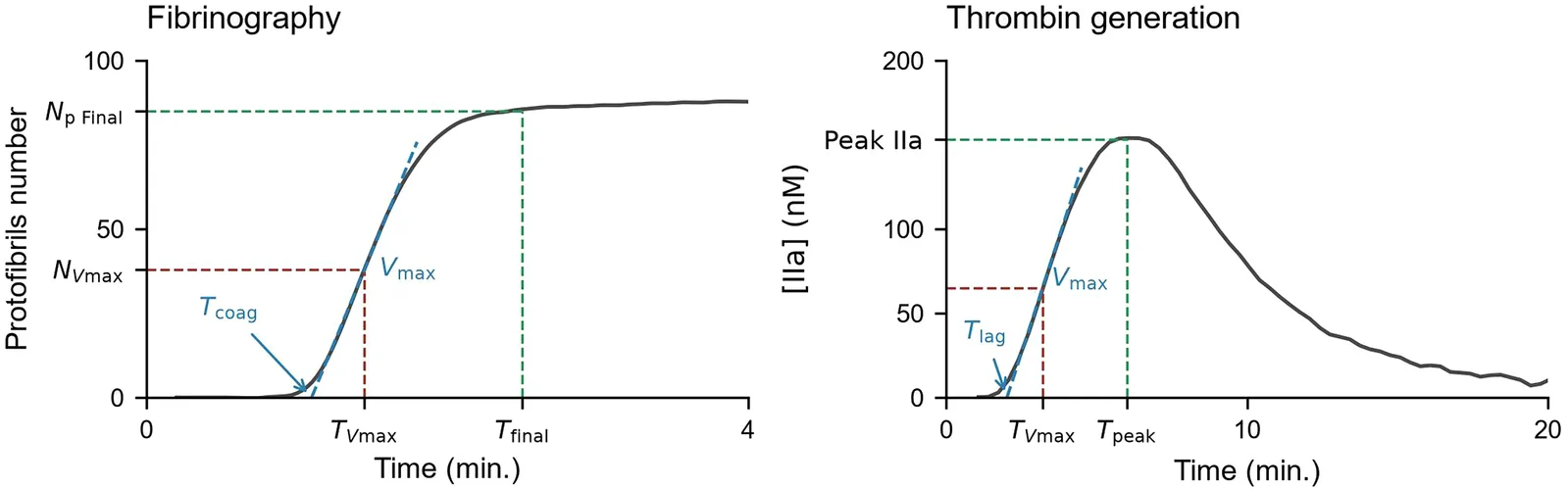
Left: Typical evolution of the number of protofibrils with characteristic times and values. Right: Typical thrombin generation curve with characteristic times and values.

Regarding thrombin generation test (TGT), the lag time (*T*_lag_), the time to peak thrombin (*T*peak), the peak concentration in thrombin (peak IIa), and the endogenous thrombin potential were calculated using the manufacturer’s software, with the addition of the maximum thrombin formation velocity (*V*_max_____IIa_) and its time of occurrence—*T*_Vmax_IIa_).

## 3 Results

### 3.1 Confocal microscopy

Figure 2 (top and bottom) shows the SME projections of the 3D confocal stacks of the fibrin networks formed from (top) normal pooled plasma clotted with varying concentrations of TF and (middle) the SME projections obtained by clotting monodisperse fibrinogen with varying amounts of purified thrombin.

**Figure 2:**
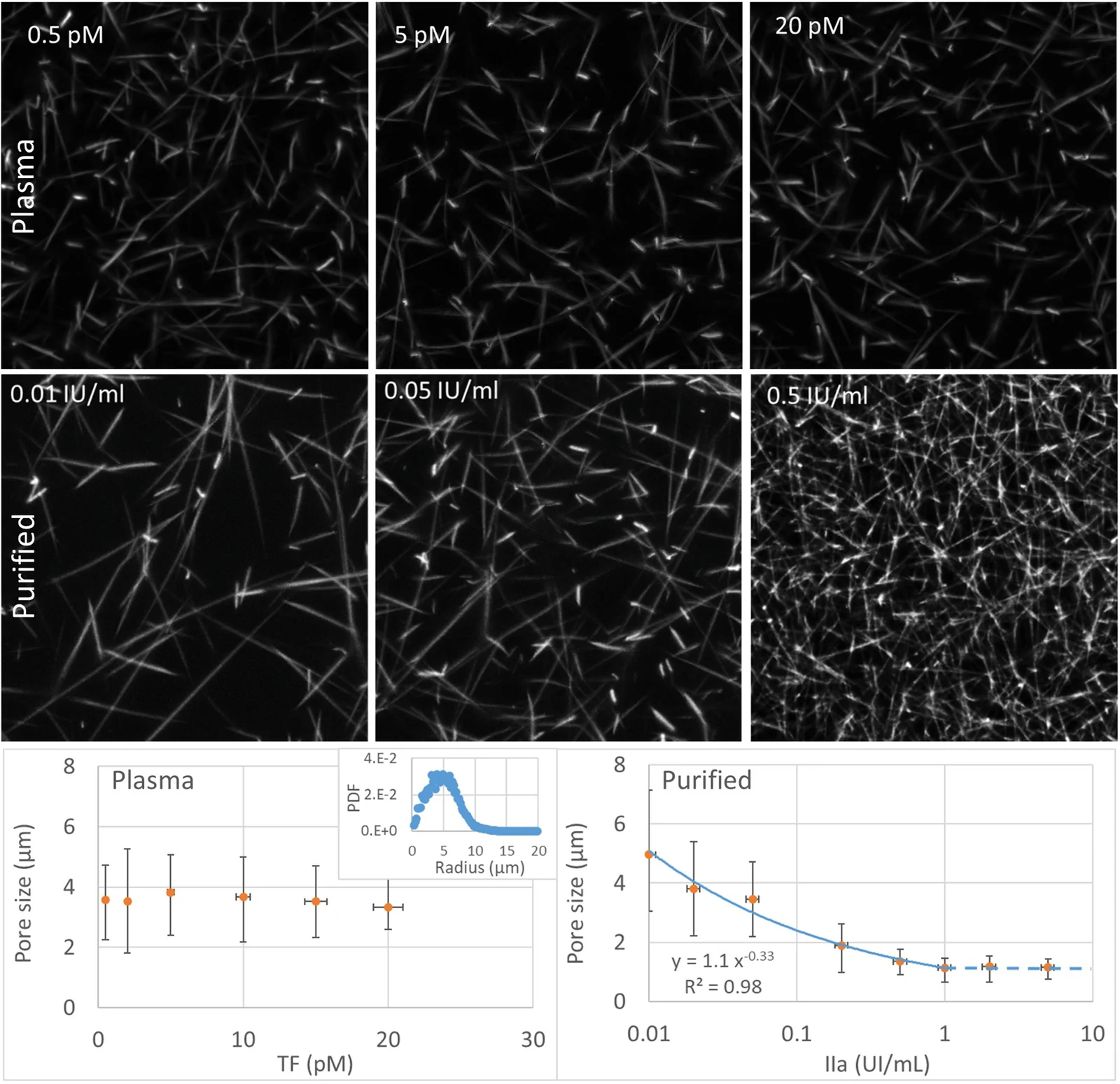
Top and middle: Smooth Manifold Extraction (SME) projections of the three-dimensional confocal imaging stacks obtained from the fluorescently labeled fibrin networks. Top: SME projections of fibrin clots formed from normal pooled plasma ([Fg] = 2.7 g/L) clotted with the tissue factor (TF) concentrations indicated on the image with a constant phospholipids concentration. Middle: SME projections obtained from monodisperse fibrinogen ([Fg] = 3 g/L) clotted with various IIa concentrations. Bottom left: Pore diameter of fibrin clots formed from normal pooled plasma clotted with varying concentrations of tissue factor. The inset shows a typical pore probability density function (PDF) which is clearly non-Gaussian. Therefore, the median of the pore size is plotted together with the error bars being the interquartile range. Bottom right: Median pore diameter of purified monodisperse fibrinogen clotted with varying concentrations of purified thrombin. Note that the [IIa] scale is logarithmic.

The first important observation was that the plasma SME projections were indistinguishable from the purified fibrinogen SME at [IIa] = 0.05 IU/mL. All SMEs displayed straight needle-like structures without discernible aggregates or tortuous configurations, with bright dots corresponding to fibers perpendicular to the projection plane. The SME projections obtained from purified monodisperse fibrinogen were very similar to those obtained previously using different batches of the same fibrinogen preparation, while they differed strongly from those obtained with standard polydisperse fibrinogen [26].

The second notable structural characteristic of normal pooled plasma clots is the absence of pore size variation in the TF range explored in this study. The median pore diameter was constant from 0.5 to 5 pM and decreased by 25% as TF increased from 5 to 20 pM (Figure 2, bottom left), well within the interquartile range. In contrast, for the monodisperse fibrinogen clotted with purified thrombin, the pore size was reduced by a factor of 5 (cf. Figure 3) when the thrombin concentration was increased from 0.01 to 1 IU/mL. Note that pore size determination above 1 IU/mL of thrombin is uncertain because the pore size is not much larger than the confocal microscope resolution [35].

**Figure 3:**
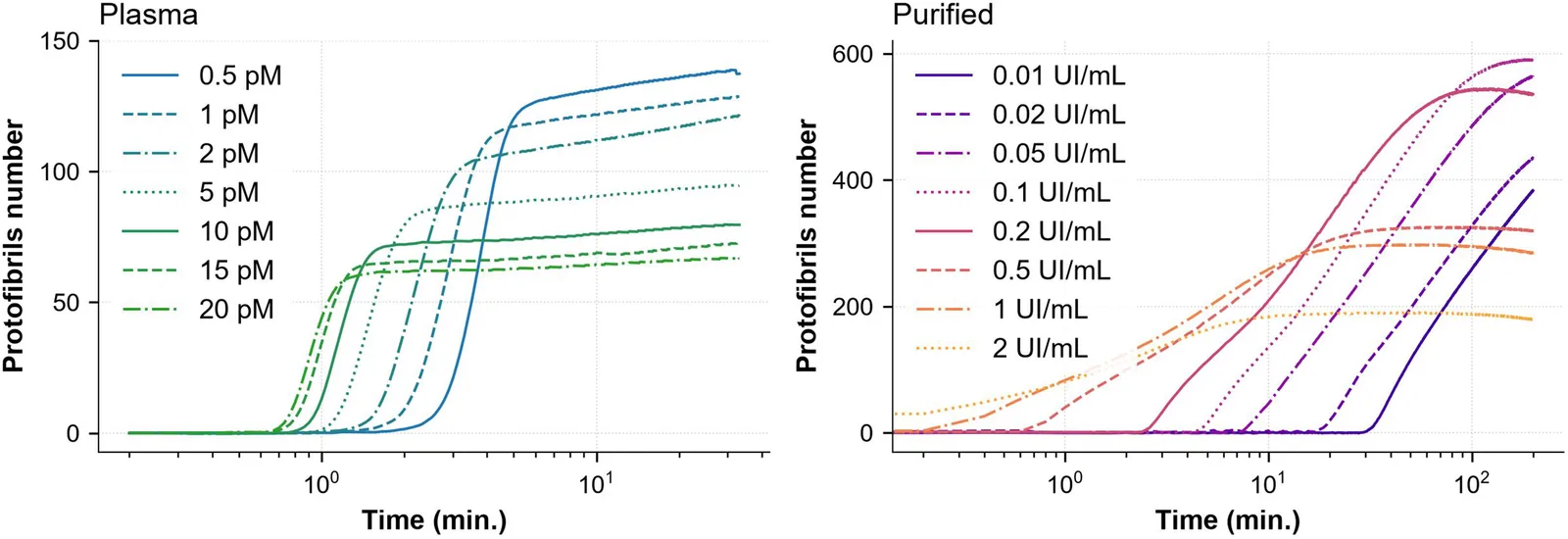
Left: Fibrinography curves obtained for *normal* pooled *plasma* ([Fg] = 2.7 g/L) clotted with tissue factor (TF) concentrations indicated on the figure with a constant phospholipid’s concentration, using a log-linear scaling. Right: Fibrinography curves for clots obtained from *purified monodisperse fibrinogen* (Fg = 3 g/L) clotted with the thrombin concentration indicated in the legend using log-linear scaling.

### 3.2 Fibrinography

#### 3.2.1 Qualitative temporal evolution of the number of protofibrils

In addition to the microstructural characterization, we examined the clot nanostructure and its formation using fibrinography. Figure 3 shows the temporal evolution of the average number of protofibrils per fiber obtained from the pooled normal plasma samples (left) and monodisperse fibrinogen (right) on a logarithm-linear scale. In both cases, the curves exhibited typical sigmoidal shapes.

For both plasma and purified fibrin clots, the clotting time and maximum number of protofibrils decreased with the increase in the control parameter. However, the clotting time range was considerably wider for purified fibrinogen than for plasma, ranging from barely measurable (∼15 seconds) to >20 minutes. In the following paragraph, to conduct a thorough comparison of fibrin nanostructure and formation in both the plasma and purified systems, the characteristic parameters (*T*_c_, *V*_max_, and *N*_Final_) were extracted from these temporal curves, as shown in Figure 1.

#### 3.2.2 Fibrinography characteristic parameters

Fibrinography characteristic parameters were plotted as a function of [TF] for plasma samples (Figure 4, right) or [IIa] for purified fibrinogen (Figure 4, left).

**Figure 4:**
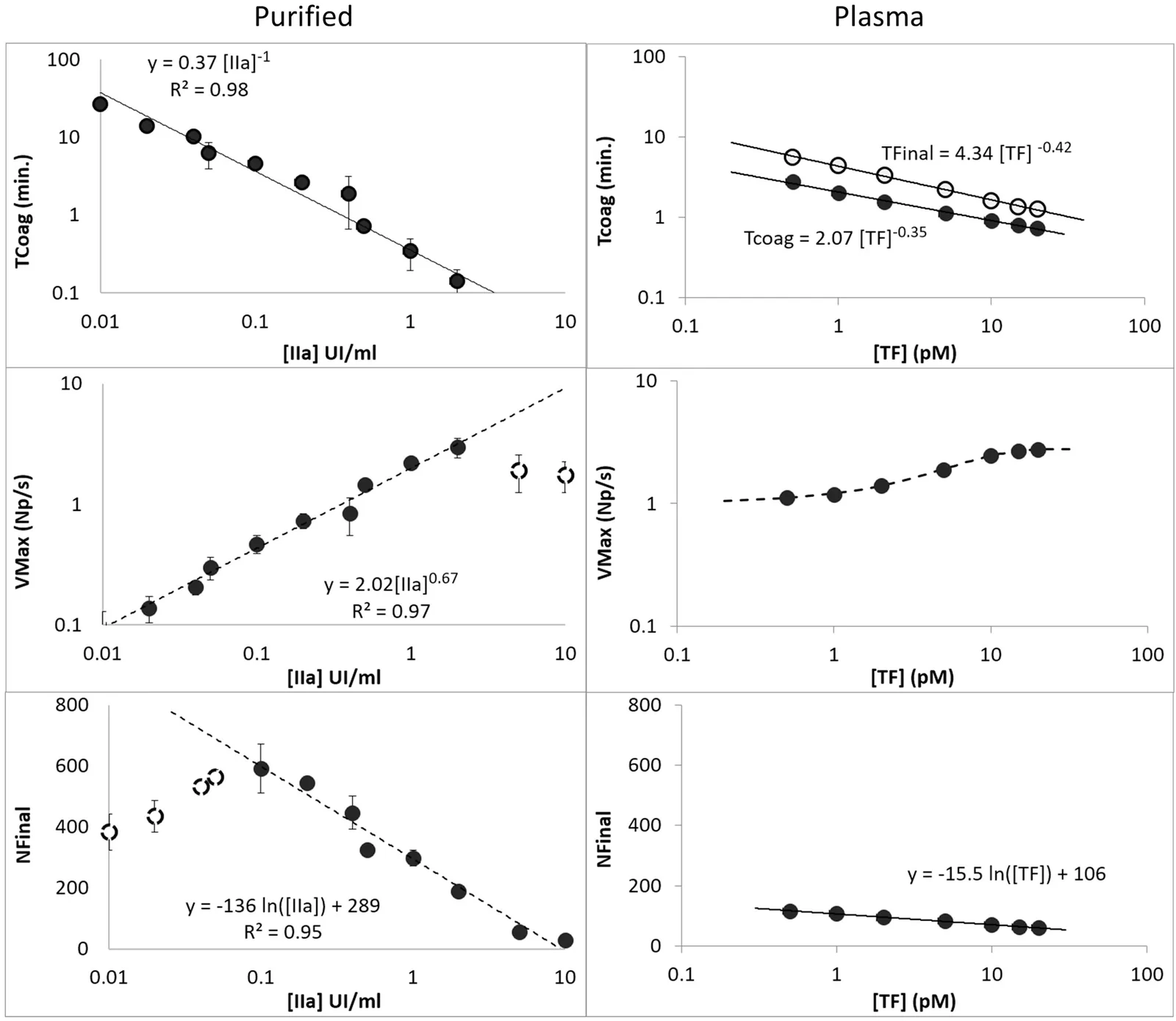
Left: Fibrinography parameters obtained for purified monodisperse *fibrinogen* clotted with various quantities of thrombin. Parameters are plotted vs [IIa] in log scale. Dashed circles correspond to the values of fibrinography parameters for which the plateau in the number of protofibrils (Np) was never reached, or when the temporal resolution was not sufficient. Right: Fibrinography parameters for clots obtained from normal pooled *plasma* clotted with varying amount of tissue factor (TF). Parameters are plotted against [TF] in log scale.

For normal pooled plasma samples, the characteristic parameters exhibited strictly monotonic behavior. Both *T*_c_ and *T*_Final_ decreased with a power law [TF]^0.4^ and decreased by a factor of 4 over the range of [TF] values studied. *V*_max_, the maximum velocity of protofibril formation, increased by a factor of 3, with a distinct sigmoidal shape, and the maximum number of protofibrils gradually decreased by 50% following a logarithmic function.

In purified samples, at the lowest [IIa] (0.01-0.1 IU/mL), the number of protofibrils did not reach a plateau after more than 3 hours of clotting (200 minutes). At the highest [IIa] (>2 IU/mL), clotting occurred on a timescale similar to the temporal resolution of the experiment. The corresponding experimental values are plotted as dashed circles in Figure 4 (bottom-left). In this figure, the number of protofibrils decreased by approximately two orders of magnitude, with [IIa] increasing from 0.1 to 5 IU/mL and best fitted using a logarithmic law. *T*_*V*max_ decreased by three decades and followed the expected 1/[IIa] law with an excellent correlation, as previously observed using single-wavelength turbidimetry [36]. Finally, the maximum velocity increased by one order of magnitude, following a two-thirds power law.

#### 3.2.3 Plasma samples: thrombin generation

The thrombin generation curves obtained from normal pooled plasma displayed the expected evolution over time (Figure 5, top left panel). More specifically, increasing [TF] (Figure 5, top right) resulted in a clear decrease in the lag time, *T*_*V*max_, and time to peak. The increase in [TF] was also associated with a large increase in peak thrombin (Figure 5, bottom left) and an increase in the maximum thrombin production velocity, which was proportional to [TF]. As expected in normal plasma, the endogenous thrombin potential was essentially independent of [TF] [37].

**Figure 5:**
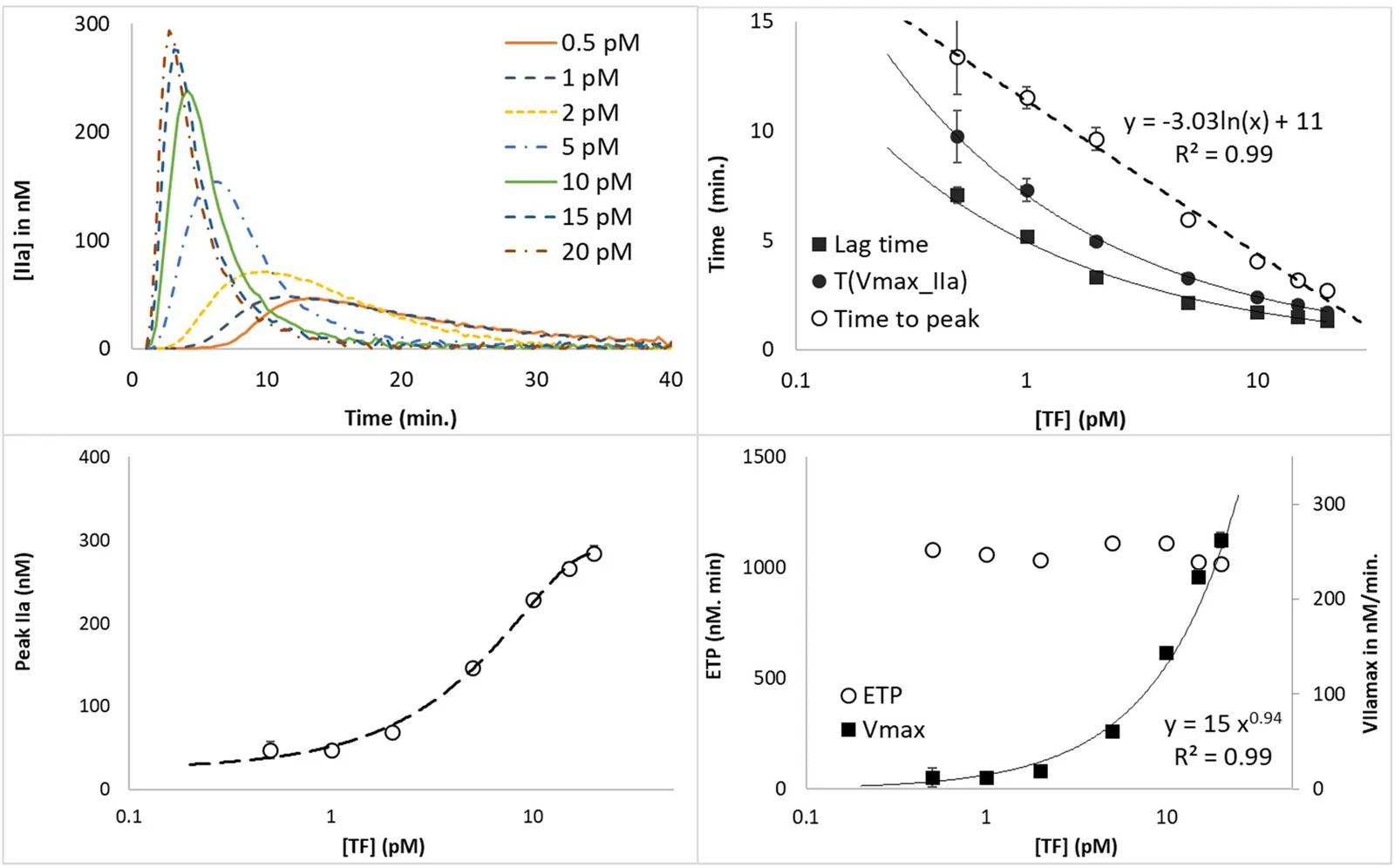
Top left: Calibrated automated thrombinography curves obtained for normal pooled plasma clotted with various [TF]. Remaining figures: evolution of the main thrombinography parameters as a function of [TF]. The [TF] (horizontal) scale is always logarithmic while the vertical axis is always linear. ETP, endogenous thrombin potential.

## 4 Discussion

### 4.1 Morphology and structure of the clots at the microscopic scale

The first important result is that the *qualitative* morphology of fibrin networks formed from monodisperse fibrinogen and purified thrombin is indistinguishable from that formed from normal pooled plasma clotted with TF. In both cases, straight, needle-like fibers were observed, which is in agreement with the century-long literature on plasma clots since the first ever description of fibrin microscopic morphology used the same words, referring to fibrin as a “crystalline gel” made of “spindle-shaped needles” [[38], [39], [40]]. In our images (Figure 2), no large isolated bright spots or tortuous structures were observed, conversely to what has previously been observed with standard fibrinogen preparations possessing a significant quantity of aggregates [23,26]. This suggests that native fibrinogen in plasma behaves as an aggregate-free population under clotting conditions and that the polydispersity and aggregates observed in the purified fibrinogens used in most studies [26] are a consequence of the purification process. Therefore, caution should be exercised when extrapolating results obtained from polydisperse fibrinogen to plasma.

Conversely, the *quantitative* structure as a function of the control parameters ([TF] or [IIa]) was very different. The average pore diameter in TF-clotted normal pooled plasma clots was constant from 0.5 to 5 pM and decreased by 25% from 5 to 20 pM. This decrease is consistent with the confocal images obtained at 1 and 10 pM of TF in pooled plasma [41]. In the purified system, when IIa was varied from 0.01 to 5 IU/mL (Figure 2), the pore diameter changed by a factor of 5. This large reduction in pore size in the purified system is in agreement with the 2-fold reduction obtained recently for IIa varying between 0.1 and 1 IU/mL [29] as well as in plasma clotted with thrombin [28,29,40].

### 4.2 Structure of clots at the nanoscale: fibrinography

The structural characteristics of the *purified fibrinogen–thrombin* clots were strongly dependent on [IIa]. A 100-fold increase in [IIa] (0.01-1 IU/mL) resulted in a decrease of ∼30-fold in the final number of protofibrils (*N*_Final_), in agreement with the literature [21,28,31,42].

Conversely, the nanostructure of *TF-triggered plasma clots* showed comparatively small changes. With [TF] varying by 40-fold, the maximum number of protofibrils showed a significant but small decrease (2-fold), which is the smallest variation, by a large margin, of all characteristic parameters. This result is in excellent agreement with, to our knowledge, the only directly comparable work, where [TF] was varied in a similar range while holding the fibrinogen concentration constant and measuring turbidity [11]. Unfortunately, the recent results in plasma from Riesman et al. [29,43] studies are not comparable since TF and fibrinogen were varied simultaneously to maintain the Fg/TF ratio constant [29], together with a large minimum amount of TF (30 or 75 pM). Finally, a less direct comparison with thromboelastography can be made because viscoelastic properties are strongly correlated with the fibrin structure. Our result is then also in agreement with the observed weak dependence of the maximum amplitude in [TF] [44].

In both the purified and plasma systems, the variations in *N*_Final_ and pore size ξ were consistent with the expected scaling argument relating fibrinogen concentration, the final number of protofibrils, and pore size. Indeed, *N*_Final_/([Fg] × $\xi^{2}$) remained constant, which shows that the different measurements are coherent within each system [26].

A direct comparison of the nanostructure obtained in both systems for similar pore sizes (3-4 μm) showed a 6-fold higher number of protofibrils (∼600 vs ∼100) for the purified system. Consequently, it appears impossible to replicate the complete architecture (micro- and nanostructure) of plasma clots using a purified system under physiological conditions (pH 7.4, 300 mOsm). A first possible explanation may be the absence of many proteins in the purified system compared with plasma. However, the nanostructure of IIa-clotted plasma clots has been shown to depend much more strongly on the IIa concentration than observed here with TF [21,28,31,40]. For instance, the number of protofibrils decreased by 6-fold with increasing [IIa] from 0.01 to 2 IU/mL [28], while the diameter decreased by a factor of 2, which is comparable with the diameter variation obtained recently under similar conditions [29]. A second possible explanation for the robustness of the architecture with the variation in [TF] may lie in the nonlinear relationship between [TF] and thrombin generated in plasma. As shown in Figure 5 (bottom, left), the peak [IIa] as a function of [TF] is a sigmoid as it increases from 50 to 240 nM when TF increases from 1 to 10 pM. Since the specific activity of the purified thrombin used for the purified system was approximately 4000 IU/mg, 1 IU/mL is equivalent to 10 nM of IIa. Then, the peak IIa variation in the plasma system should be equivalent to a variation of 5 to 24 IU/mL of thrombin in the purified system, which would clot fibrinogen within a few seconds (Figure 4, left, top), which is clearly not what is observed in plasma. This is not surprising, since it has long been known that the largest part of thrombin generation is produced after fibrin formation [45].

The present structural results suggest that the thrombin burst and thus the clotting cascade play a crucial role in obtaining a final architecture that appears to be fairly robust vs TF concentration, since the number of protofibrils only changed by 2-fold while the TF concentration increased by 40-fold. Interestingly, this adds weight to the concept of a “normal clot” [9]. In the following section, we investigate whether thrombin generation kinetics—rather than plasma composition—can quantitatively account for the observed structural robustness.

### 4.3 Kinetics of fibrin structure and thrombin generation

Ideally, to understand how thrombin kinetics may control the architecture of TF-triggered plasma clots, one would try imposing a controlled thrombin evolution during plasma clotting and measure the resulting architecture. However, this direct experimental approach is physically self-defeating, regardless of technical feasibility. Indeed, delivering varying quantities of thrombin homogeneously throughout a reaction volume that is transitioning from a liquid to a solid state would strongly perturb the polymerization process itself. If a flow condition was used, it would mechanically disrupt the forming clot, while the use of controlled diffusion would generate large concentration gradients, fundamentally altering in both cases the very phenomenon under investigation. Another possibility would be to try predicting the final fibrin architecture (in our case, the pore size and the final number of protofibrils) from [TF] and [Fg] using a mathematical model of fibrin polymerization, where a thrombin generation model [12,14,46,47] could be used as an input, and then compare the model with experimental data. Unfortunately, as recently reviewed [48], current fibrin polymerization models either lack thrombin as an explicit variable [49] or have a fixed thrombin concentration during the reaction [[50], [51], [52]], which is not compatible with the actual coagulation cascade, where thrombin concentration varies by orders of magnitude [45].

Two alternative strategies can be proposed: the first is to use the thrombin evolution determined experimentally from TGT together with the structure of clots formed from purified thrombin and fibrinogen at characteristic times to predict the clot structure in plasma at those times and compare it with the structural data. However, when comparing Figures 4 (top left) and 5 (top right), it appears that the lag time is close to the end time in fibrinography (*T*_Final_), which means that a large part of fibrin polymerization is already completed before TGT detects the first “traces” of thrombin, an observation that is in agreement with the study by Xin et al. [11], rendering this approach impossible with current thrombinography methods. The reverse approach, that is, predicting [IIa] at specific times using structural data, is feasible because the structural evolutions in both the plasma and purified clots are known on timescales compatible with the TGT data. Although this method does not predict the structure from thrombin evolution, it allows for the determination of whether the observed plasma architecture is the result of clotting fibrinogen with a TGT-like evolution of thrombin or whether other factors are crucial for plasma fibrin polymerization.

The prediction of thrombin concentrations in TF-clotted plasma at characteristic times requires knowledge of the relationships obtained in the purified system between these characteristic times, associated structural parameters, and thrombin concentrations. These were obtained by inverting the fitted relationships between the parameters and the thrombin quantity obtained for the purified clots, as shown in Figure 3, Figure 4, Figure 5. At the clotting time (*T*_coag_), the thrombin quantity was calculated from the purified experiments as ${{II}_{a}}^{T\text{coag}}=\frac{0.366}{T_{\text{coag}}}$, whereas at the time of maximum velocity of protofibril aggregation (*T*_*V*max_), the calculated thrombin quantity was ${{II}_{a}}^{V\;\max}={\left(\frac{V\;\max}{121}\right)}^{1.5}$. The thrombin concentration at the time of maximum number of protofibrils (*T*_Final_) was ${{II}_{a}}^{N\text{Final}}=\exp\left[\frac{-N\text{Final}-289}{136}\right]$. Finally, because the micronic structure is determined at the gel point, the amount of thrombin at the gel point is ${{II}_{a}}^{Gp}=1.89{\left(\xi \right)}^{-3.1}$, where ξ is the pore size. Note that the gel point time *T*_gp_ was not determined experimentally in this study. Using these expressions, the thrombin concentrations in plasma at *T*_c_, *T*_*V*max_, and *T*_95%_ were calculated from the *T*_c_, *V*_max_, and *N*_Final_ values obtained from fibrinography in plasma. The (*T*_c_, *IIa*^*T*c^), (*T*_*V*max_, ${{II}_{a}}^{V\;\max}$), and (*T*_Final_, ${{II}_{a}}^{\text{Final}}$) values are reported on the corresponding CAT curves in Figure 6.

**Figure 6:**
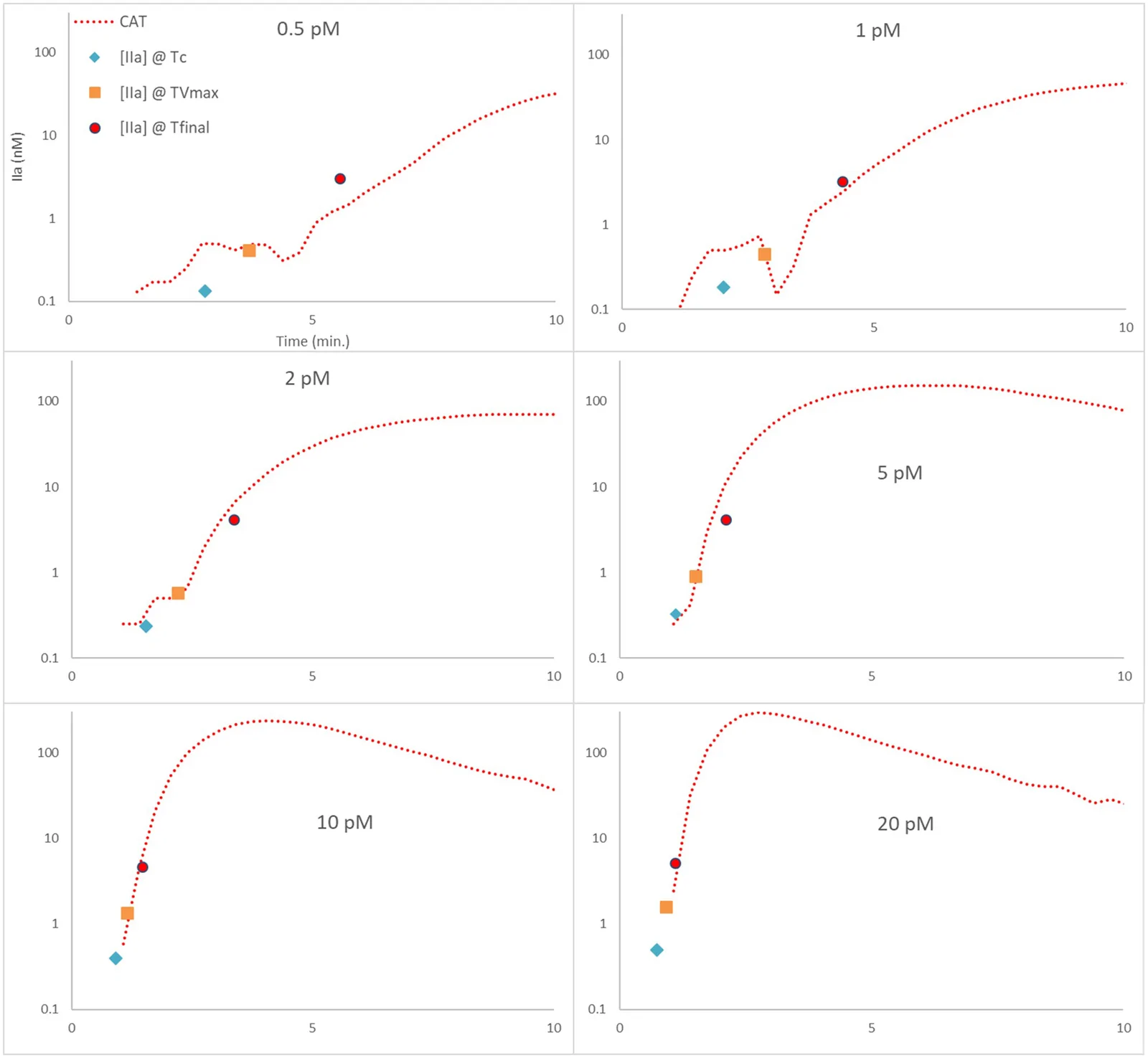
*Experimental thrombin generation curves (calibrated automated thrombinography [*CAT], triplicate averages) together with thrombin concentration predictions at T_*c*_*,* T_Vmax_, and T_Final_. Note the linear timescale and logarithmic [IIa] scale. The CAT curves are the same as those in Figure 5 top left.

The agreement between the predictions and measurements is remarkable, spanning two decades of [TF] and obtained on the exponentially growing portion of the thrombin generation curve. Furthermore, the predictions use directly, without any adjustable parameter, the relations established from the purified system. This provides two lines of evidence that thrombin generation kinetics is the main determinant of structural clot robustness. First, this prediction success would not be expected if plasma-specific factors were dominant. Second, published data consistently show that IIa-triggered plasma clots exhibit strong [IIa]-dependent structural changes, the magnitude of which is similar to that observed in the purified system [21,28,32,41]. In contrast, as shown here, TF-triggered plasma clots exhibit remarkable structural robustness. Since plasma pool composition is essentially constant, this robustness cannot be attributed to plasma composition and must instead reflect the kinetic profile of thrombin generation. Taken together, this shows that, in normal plasma, the characteristics of the thrombin burst control fibrin structuring at the molecular, nano-, and micro-scales. This produces clots with a structure that is robust to variations in [TF] in the range studied here. Interestingly, this complements the different roles of thrombin described by Kenneth Mann in his seminal paper “What is all that thrombin for” [45].

This structural robustness has potential practical implications, as it paves the way for the further development of structural biomarkers. Given the clot structure constancy in normal plasma while varying [TF], it may be feasible to establish a range of normal structural values. However, the present data are derived from a single pooled plasma preparation, which averages out interindividual biological variability. Also, whether comparable robustness is observed across individual donors remains to be determined, together with evaluation in pathologic populations. In addition, the excellent reproducibility of the fibrinography assay [32] suggests the possibility of automating and standardizing an assay for determining fibrin clot structure.

The present study has a major limitation: it is a fundamental mechanistic investigation performed on a single pooled normal plasma. Therefore, substantial additional work, including cohort-based studies with individual donor samples, pathologic plasma samples, and correlation with clinical outcomes, is necessary to build upon these fundamental findings.

## 5 Conclusion

The current study is a basic science investigation of the structure of TF-triggered frozen normal plasma and purified fibrinogen and thrombin. Contrary to intuition, the results showed that the clot structure in plasma was fairly robust, meaning that the structure at different scales depended only weakly on [TF]. The [IIa] concentrations at characteristic times were predicted from the purified and plasma structural results and compared successfully with thrombinography data. This suggests that the observed robustness of the structure of TF-triggered plasma clots can be traced back to the unique functional shape of thrombin generation. This highlights the crucial interplay between thrombin generation and fibrin structuring necessary for forming a "perfect clot” [9], demonstrating that both sets of information are mandatory for understanding plasma clotting. The stability of this TF-triggered plasma clotting vs a large change in the trigger concentration is reminiscent of the robustness concept developed in the systems biology literature.

In pathologic states, we expect that this interplay will be disturbed in many different ways; therefore, obtaining both thrombin generation and fibrin structuring in the same sample should provide valuable insights into the disturbed mechanisms. Therefore, further studies conducting similar experiments with patient plasma samples and comparing fibrinography and thrombinography with clinical outcomes will be of great interest.
